# 

**Published:** 1884-02

**Authors:** 


					﻿±'£iJUX(,UA.£&X, XOO4.
THIRTY-SHFTST YEAR.
IIAJJ/S
JOURNAL
OF
MXJoJ^Aj JL JEM
Guaranteed Circulation 200,000 Copies in 12 Months.
E. H. GIBBS, A.M., M.D., Editor,
No. 21 CLINTON PLACE, EIGHTH STREET, NEW YORK.
TEEMS: $1 a Year.
Smale number, 10 cts.
				

